# Effect of Dystocia Duration on the Placental Health in Canines

**DOI:** 10.3390/life16020349

**Published:** 2026-02-18

**Authors:** Romina Gisele Praderio, Mauricio Javier Giuliodori, Rodolfo Luzbel de la Sota, María Alejandra Stornelli

**Affiliations:** 1 Instituto de Investigaciones en Reproducción Animal (INIRA), Facultad de Ciencias Veterinarias, Universidad Nacional de La Plata, La Plata 1900, Argentina; mauriciog@fcv.unlp.edu.ar (M.J.G.); luzbel@fcv.unlp.edu.ar (R.L.d.l.S.); astornel@fcv.unlp.edu.ar (M.A.S.); 2 Consejo Nacional de Investigaciones Científicas y Técnicas (CONICET), Godoy Cruz 2290, CABA 1425, Argentina

**Keywords:** dogs, placenta, dystocia duration, obstetric evaluation

## Abstract

The study aimed to determine whether placental lesions differ according to the duration of dystocia. Forty-seven placentas were obtained from 18 bitches that underwent emergency cesarean sections. For descriptive purposes, the cases were classified into four groups based on the duration of dystocia: Group A, up to 6 h; Group B, 6–11.9 h; Group C, 12–24 h; and Group D, more than 24 h. Forty-seven placentas were studied. Both macroscopic and microscopic characteristics were evaluated in each placenta. Descriptive data were presented, and logistic and multinomial regression models were used to assess whether dystocia duration (in hours) is associated with the presence and severity of placental macro- and microscopic lesions. An hour increment over the mean in the duration of dystocia showed a non-significant trend to increasing the presence of macroscopic necrosis (OR: 1.11, *p* = 0.09) and mineralization (OR: 1.10, *p* = 0.06), and it also increased the severity of macroscopic congestion (OR: 1.44; *p* = 0.01) and showed a non-significant trend to increasing the severity of polymorphonuclear neutrophil infiltrate (OR: 1.18; *p* = 0.06). These findings highlight the importance of early obstetric intervention in all cases of dystocia to minimize fetal hypoxia and improve neonatal outcomes. Moreover, the placenta could serve as a biomarker for fetal distress, as the presence of severe lesions indicates an increased risk for reduced neonatal survival.

## 1. Introduction

Neonatal viability is influenced by several factors, including litter size, parturition type, and maternal health [1,2]. Early neonatal mortality is defined as the death of neonates within the first seven days of life, whereas perinatal mortality also includes the stillbirths [3,4]. In dystocic births, neonatal and perinatal mortality increases significantly, primarily due to hypoxia suffered by the fetus inside the uterus during prolonged labor, leading to fetal depression [4,5].

The fetus depends entirely on the placental oxygen exchange through the umbilical cord and on adequate maternal respiration and circulation [6]. During normal parturition, uterine contractions cause intermittent or transient hypoxia to the fetus [7,8,9,10]. Under adverse conditions, such as dystocia, these periods of hypoxia are more intense and prolonged, reducing placental perfusion and oxygen delivery to the fetus [1]. This reduced placental perfusion can result in severe hypoxia, acidosis, and, finally, fetal death. Recent studies of canine placentas obtained by cesarean sections or deliveries have reported macroscopic and microscopic alterations [2,11,12,13,14]. In human medicine, placental hypoxia has been linked to the development of placental lesions, including laminar necrosis and microscopic chorionic pseudocysts [15,16]. To our knowledge, limited data are available from canine studies examining the relationship between the duration of labor and placental injury. Recent studies have shown an association between placental lesions and fetal and neonatal mortality [2,14]. However, so far, the lesions and degrees of placental injury according to the duration of labor or dystocia are not well established. Therefore, studies on this topic are necessary to understand how dystocia duration affects placental integrity and its relationship to fetal hypoxia, thereby enabling improved obstetric management in dogs. Hence, the objective of this study was to determine whether there are differences in placental lesions based on the duration of dystocia. We hypothesized that the severity of placental lesions increases as the duration of dystocia advances.

## 2. Materials and Methods

### 2.1. Design and Animals

A transversal observational study was conducted on 18 bitches, both mixed-breed and purebred, aged 1.5 to 10 years, that presented with dystocia at the Veterinary Teaching Hospital of the Facultad de Ciencias Veterinarias, Universidad Nacional de La Plata (UNLP), La Plata, Buenos Aires, Argentina. All the bitches were submitted to a clinical examination, followed by an emergency cesarean section. Seventy-two percent of the bitches (n = 13) were primiparous and 28% (n = 5) multiparous. Dystocia was defined as the inability to deliver without assistance. When a bitch strained for more than an hour and no expulsion occurred, the dystocia was classified as obstructive dystocia. Bitches with eutocic labor or elective cesarean sections were excluded from this study. The bitches included had no history of uterine surgeries, nor were they suffering from systemic diseases at the time of the experiment. None of the bitches were obese or underweight based on clinical examination.

The study was approved by the Institutional Animal Care and Use Committee of the FCV-UNLP (122-2-22P), and was conducted with informed consent, read and signed by each owner, before each bitch was included in the study.

For descriptive purposes, the bitches were divided into four groups according to the dystocia length recorder and reported by the owners:

Group A: up to 6 h of dystocia.

Group B: 6–11.9 h of dystocia.

Group C: 12–24 h of dystocia.

Group D: more than 24 h of dystocia.

Table 1 summarizes the data for the animals included in each group.

### 2.2. Samples

Forty-seven placentas were obtained from 18 bitches that underwent emergency cesarean section. Placentas were transported to the laboratory, separated from the extraplacental membranes, and washed with physiological saline solution. Then, macroscopic and microscopic lesions were assessed.

Macroscopic congestion was characterized by the presence of violet-colored areas visible in the placental girdle, and macroscopic necrosis was characterized by yellow ochre-colored areas visible in the placental girdle. Microscopic congestion was characterized by excessive accumulation of blood within placental blood vessels. Microscopic necrosis was characterized as loss of structural detail in placental areas, accompanied by eosinophilic cytoplasm and nuclear changes, including pyknosis, karyorrhexis, and karyolysis, or by the absence of nuclei. The presence of polymorphonuclear leukocytes characterized the inflammatory infiltrate. Mineralization was characterized by basophilic stippling in necrotic cells, indicative of calcium salt deposition.

The placental surface was evaluated for macroscopic congestion and necrosis using a 4-point scale (0 = absent; 1 = mild, 1 lesion occupying ˂1% of the surface of the placental girdle; 2 = moderate, lesions occupying 1–2.5% of the surface of the placental girdle; 3 = severe, lesions occupying >2.5% of the surface of the placental girdle or more than 4 lesions), following previous descriptions [2,11]. Then, a biopsy of 1 × 1 cm of the central region was obtained with a scalpel in each placenta and subsequently fixed in 10% buffered formalin [2]. After histological processing, the samples were embedded in paraffin, sectioned with a microtome, and stained with hematoxylin and eosin (H&E). The slides obtained were examined under an optical microscope at X400 for recorded necrosis, congestion, mineralization, and infiltration of polymorphonuclear cells at X400 in three randomly selected fields using a 3-point scale (0 = no lesion, 1 = up to 40% of the evaluated field affected, 1–3 lesions; 2 = more than 40% of the field affected, >3 lesions) [2,11]. All samples were evaluated by a single operator who was blinded to dystocia duration and stillbirth status for microscopic features, but not for macroscopic features.

### 2.3. Statistical Analysis

Descriptive data for each dystocia group (A–D) were estimated using Proc Univariate in SAS. For all the statistical analyses, placentas were nested within bitches. Logistic regression models were used to analyze the effect of dystocia duration (in hours) as a quantitative predictor on the likelihood of placental lesions (Yes vs. No), such as macroscopic congestion, macroscopic necrosis, microscopic congestion, microscopic necrosis, infiltration, and mineralization. Logistic models were run in Proc Glimmix of SAS with a binomial distribution and a logit link function. In logistic regression, odds ratios for quantitative predictors such as dystocia duration (in hours) indicate how the odds of each lesion change per 1 h increment above the dystocia duration. Multinomial logistic regression models were used to analyze the effect of dystocia duration (in hours), as a quantitative predictor, on the severity of the above placental lesions (absent vs. degree 1 vs. degree 2 vs. degree 3 for macroscopic lesion, and degree 0 vs. degree 1 vs. degree 2 for microscopic lesions). Multinomial models were run in Proc Glimmix of SAS with a multinomial distribution and a glogit link function. In multinomial regression, the odds ratios for quantitative predictors such as dystocia duration (in hours) indicate how the odds for each lesion grade change per increment of 1 h above the mean dystocia duration. Goodness of fit in logistic and multinomial regression was assessed by the Likelihood Ratio test (the smaller the value, the better the fit), and Pearson Chi-Square/DF (the closer to 1, the better). Additionally, visual inspection of residual plots was performed. All analyses were conducted in SAS^®^ On Demand for Academics 3.81 Enterprise Edition (SAS Institute Inc., Cary, NC, USA). Statistical significance was set at *p* < 0.05, and a trend for significance at *p* < 0.10.

## 3. Results

### 3.1. Descriptive Data

**Figure 1:**
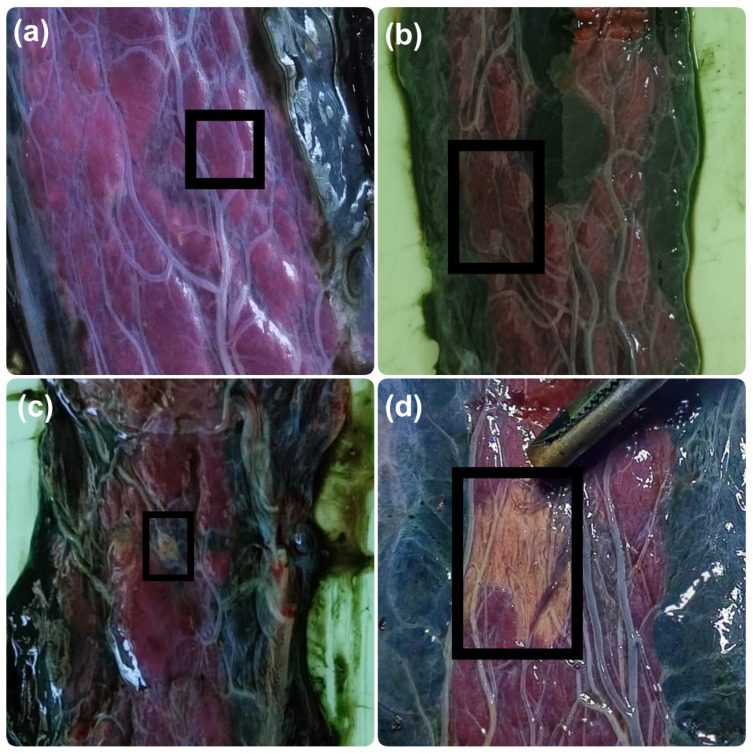
Macroscopic placental lesions. Representative images show congestion grade 1 (**a**) and grade 3 (**b**) and necrosis grade 1 (**c**) and grade 3 (**d**). Lesions were scored as: 0 = no alteration, 1 = mild, 2 = moderate, and 3 = severe [2,11].

**Figure 2:**
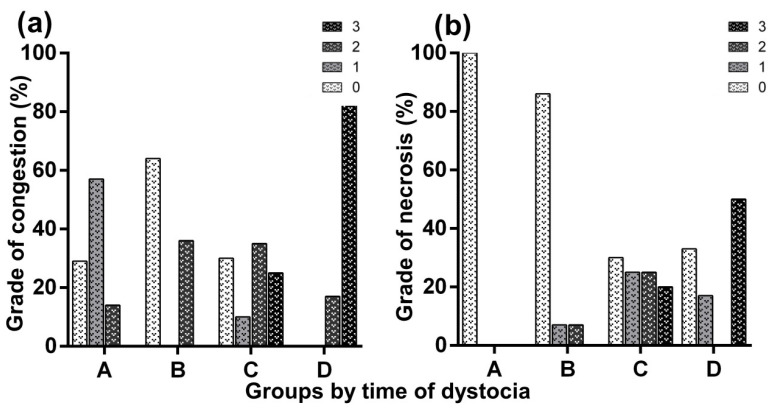
Distribution of macroscopic changes observed in canine placentas at different durations of dystocia (A: up to 6 h, B: 6.1–11.9 h, C: 12–24 h, D: more than 24 h). (**a**) Grades of macroscopic congestion; (**b**) grades of macroscopic necrosis, expressed as percentages within each group. Lesions were scored as: 0 = no alteration, 1 = mild, 2 = moderate, and 3 = severe [2,11].

**Figure 3:**
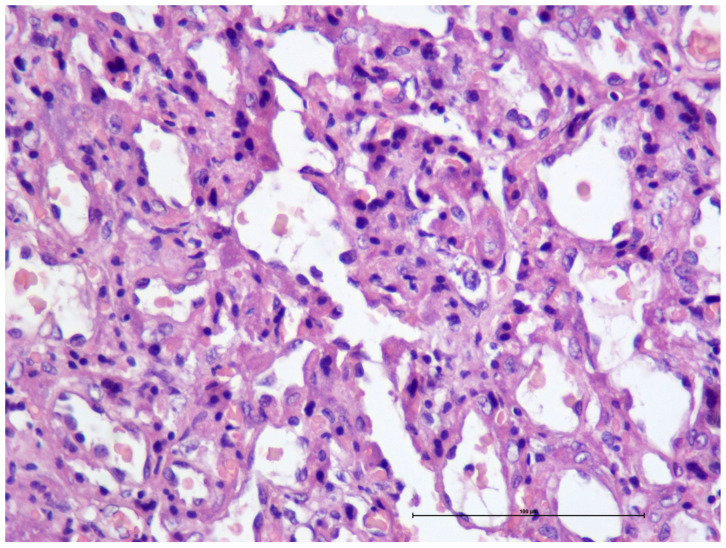
Histological image of normal placenta stained with hematoxylin and eosin (H&E). Magnification X400 [scale bar 100 μm].

**Figure 4:**
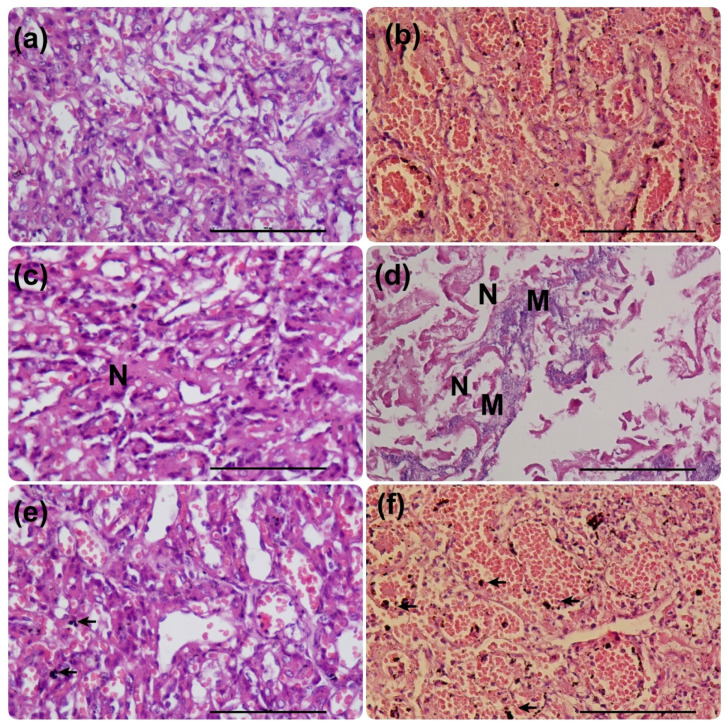
Histological images of placental lesions stained with hematoxylin and eosin (H&E). Magnification X400 [scale bar 100 μm]. Representative images show congestion grade 1 (**a**) and grade 2 (**b**); necrosis grade 1 (**c**) and grade 2 with mineralization (**d**); and infiltration of polymorphonuclear cells grade 1 (**e**) and grade 2 (**f**). N, necrosis; M, mineralization. Black arrows indicate polymorphonuclear cells. Lesions were scored as: 0 = no lesion; 1 = up to 40% of the evaluated field affected, 1–3 lesions; and 2 = more than 40% of the field affected, >3 lesions [2,11].

**Figure 5:**
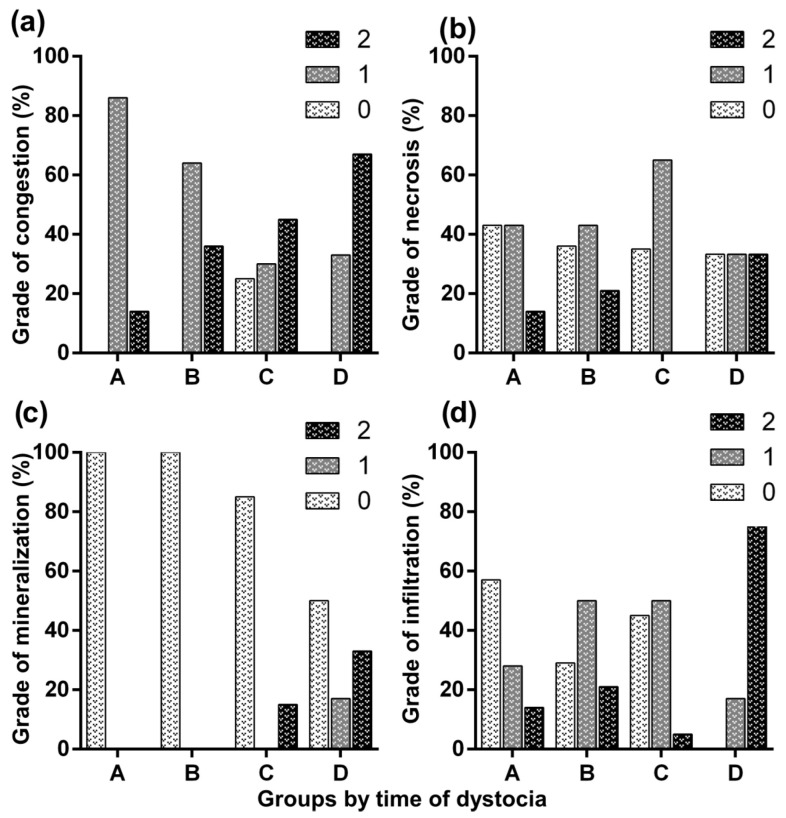
Distribution of microscopic changes observed in canine placentas at different durations of dystocia (A: up to 6 h, B: 6.1–11.9 h, C: 12–24 h, D: more than 24 h). (**a**) Grades of microscopic congestion; (**b**) grades of microscopic necrosis; (**c**) grades of mineralization; and (**d**) grades of infiltration of polymorphonuclear cells, expressed as percentages within each group. Lesions were scored as: 0 = no lesion; 1 = up to 40% of the evaluated field affected, 1–3 lesions; and 2 = more than 40% of the field affected, >3 lesions [2,11].

Of the 18 bitches included in this study, three belonged to Group A, four to Group B, seven to Group C, and four to Group D. Seven placentas belonged to females of Group A, 14 to Group B, 20 to Group C, and 6 to Group D. These placentas corresponded to both stillborn and liveborn puppies, except in Group D, where all puppies were stillborn. The relationship between dystocia duration and neonatal outcome was not linear. The stillbirth rate was 85.7% (6/7) in Group A, 42.8% (6/14) in Group B, 60% (12/20) in Group C, and 100% (6/6) in Group D. All females included in this study, in all groups, survived the surgical procedure. Figure 1 and Figure 2 illustrate the macroscopic alterations observed in the placentas. Figure 3 shows a placenta with normal histology, and Figure 4 and Figure 5 illustrate the microscopic alterations observed. Figure 2 and Figure 5 depict pathological changes only and do not represent stillbirth percentages. Complete information is available in Supplementary Table S1.

### 3.2. Effect of Duration of Dystocia on the Placenta

According to logistic models, each one-hour increment in the duration of dystocia showed a non-significant trend toward increased risk of macroscopic necrosis (OR: 1.11, *p* = 0.09; Table 2) and mineralization (OR: 1.10, *p* = 0.06; Table 2). According to multinomial models, an increment of one hour in the duration of dystocia increased the risk of having severe macroscopic congestion (Grade 3, OR: 1.44; *p* = 0.01; Table 3) and showed a non-significant trend toward increasing the presence of abundant polymorphonuclear neutrophil infiltrate (Grade 2, OR: 1.18; *p* = 0.06; Table 3). The effect of dystocia duration on the remaining response variables was not significant (Table 2 and Table 3).

**Figure 6:**
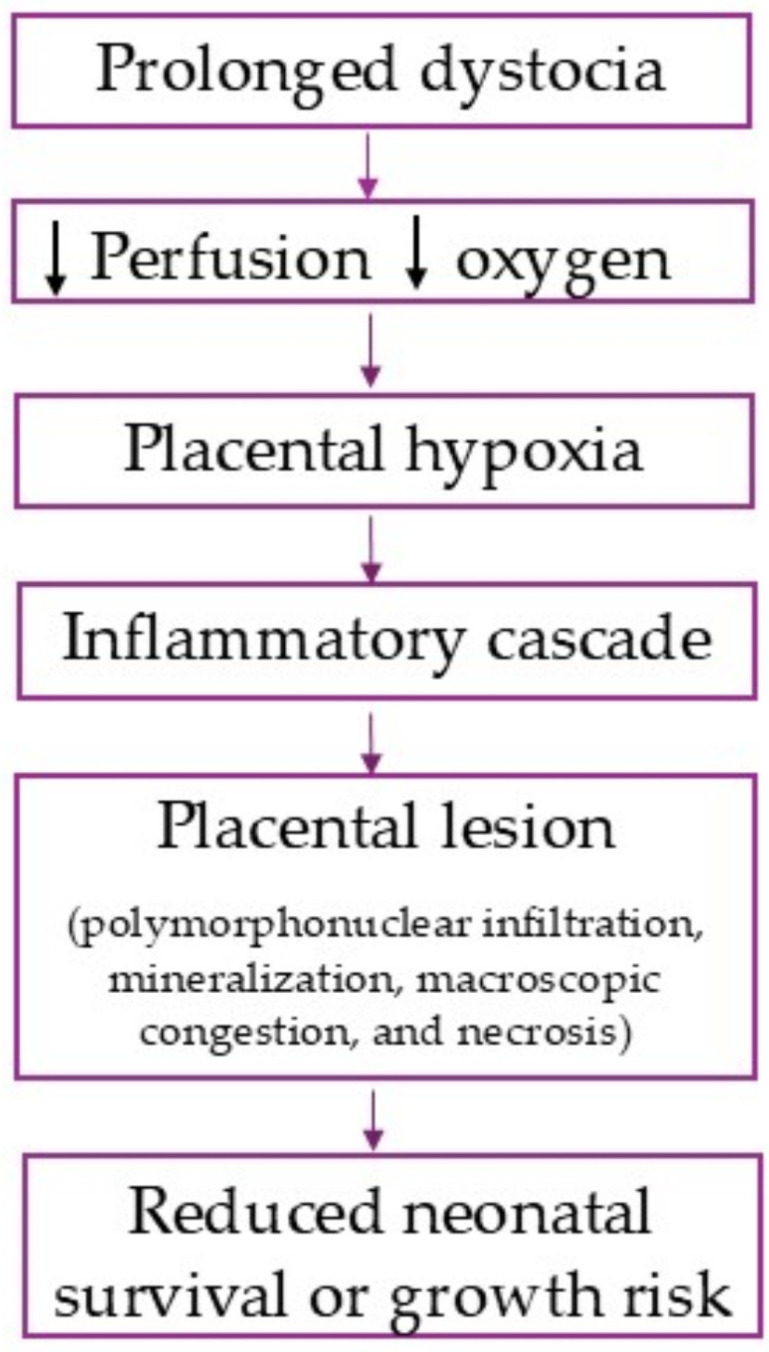
Conceptual model.

Figure 6 summarizes the proposed model of the placental hypoxia process in dystocia.

## 4. Discussion

To our knowledge, no prior studies have systematically quantified placental lesions according to reported dystocia duration in dogs. A direct relationship was observed between the duration of dystocia and the presence and severity of placental damage. As the duration of dystocia increased, the presence of macroscopic necrosis and mineralization tended to increase, and the severity of congestion and polymorphonuclear infiltration also increased. Mineralization was observed exclusively in placentas from prolonged dystocia cases. In cases lasting more than 24 h, the lesions were severe, and none of the newborns survived. Reporting these findings is important because information on placental evaluation is scarce in veterinary neonatology. During obstetric evaluation, the placentas should be examined to understand the degrees of injury associated with a specific duration of dystocia, which may help the veterinarian identify the newborns at a higher risk of neonatal mortality.

Previous studies have reported complications associated with cesarean sections, such as those related to the anesthesia of the dog, intra- and post-operative hemorrhages, coagulation problems, surgical techniques, endometritis, and uterine rupture, among others, even leading to maternal death [17,18,19]. In this research, fortunately, all the bitches survived, although the percentage of perinatal mortality was elevated in all groups. Although cesarean sections carry a considerable risk of maternal complications, prolonged delays in intervention endanger fetal survival and further complications for the bitch. Therefore, prevention is essential and should include assessing dystocia risk, educating owners, and frequent clinical and ultrasound monitoring in all pregnant females.

The results of this investigation highlight the consequences of the delayed intervention. Infiltration of polymorphonuclear cells and macroscopic congestion were observed in severe degrees in prolonged dystocia. These associations are consistent with a model in which prolonged hypoxia contributes to inflammation, leading to vascular congestion and polymorphonuclear infiltration. As indicated by previous studies, these placental alterations have been commonly documented in mild degrees in placentas from natural deliveries [2,11]. It has been reported that when these lesions are mild, there are no complications for the newborns [14]. In the current research, these lesions were observed in a high proportion of placentas; however, they were observed in mild or moderate degrees in placentas from dystocias of short duration. It is well established that the impact of acute and chronic hypoxia depends on the time of exposure [20]. The effect of high-grade lesions in the canine neonatal period remains unclear. We hypothesized that these severe lesions could indicate a poor neonatal prognosis.

In previous studies, mineralization was observed to a mild degree in placentas of bitches undergoing emergency cesarean sections, although the duration of dystocia was not specified [12,13,14,21]. Furthermore, a study also observed this lesion to a mild degree in placentas obtained via elective cesarean sections [13]. In the present study, this alteration was found to be associated with prolonged dystocias of 24 h or more and with stillborns. In humans, a severe degree of mineralization was associated with preeclampsia and fetal distress [22]. Previous research has suggested that placental mineralization is influenced by hypoxia [23,24]. Our results support these findings, since mineralization was associated with a prolonged period of hypoxia.

The severity of these lesions shows a higher probability of fetal distress, which may have further implications for neonatal health. Previous studies have demonstrated that uterine conditions have a long-term impact on the conceptus’ physiology and metabolism throughout its life [25,26]. In human medicine, there have been reports of various disabilities in children who have experienced perinatal prolonged hypoxia, including convulsions, intellectual disability, speech impairment, visual impairment, hearing impairment, neurological impairment, nutritional impairment, and respiratory impairment [27,28,29,30,31]. In canines, the long-term consequences of perinatal hypoxia have not been reported; however, they may also affect neonatal growth. A previous study found that multifocal placental necrosis was associated with poor neonatal outcomes in the early neonatal period [21]. It has recently been reported that newborns whose placentas showed macroscopic necrosis were less likely to double their weight in the first 10 days of life [2]. Although this effect was not significant for other placental lesions, the authors suggest further studies on this topic [2].

Previous studies in canines have reported that inflammatory infiltrate in placentas at term may be a common finding [2,11,12,13]. Studies in human medicine suggest that inflammation is necessary for the onset of labor [32,33]. Another study proposed that labor duration influences placental gene expression, which is associated with dysregulation of TNF, IL6, IGF1, and IGF2, highlighting the need for further research on the impact of these alterations on the mother and the neonate [34]. Taken together, these findings support the observation that placentas from prolonged dystocia exhibit an abundant inflammatory infiltrate, as observed in the current study. However, further assays are still required to determine the effects of this inflammatory response on neonatal outcomes in the postnatal period.

The incidence of stillbirths observed in this study was high in all groups (42.3 to 100%). When dystocia exceeded 24 h, the mortality rate was 100%. This finding is consistent with previous literature, which reports that the number of stillbirths in puppies increases in prolonged births [7,35]. In the Group A, the incidence of stillbirth was higher than in Group B and C. This could be related to the type of dystocia, breed differences, litter size, or pre-existing maternal or fetal compromise. In one investigation, the authors highlighted that dogs are a distinctive species characterized by a multitude of breeds and that this diversity implies a diverse metabolism, which manifests under different physiological conditions [36]. Other studies have shown an association between stillbirth rate and litter size [2,4,35]. The fact that dystocia lasts up to 6 h does not mean that it is not harmful to the female and her newborns. The present study does not imply that brief dystocia is without risk, as pre-existing conditions can increase the neonatal mortality rate. Previous studies have shown that the longer the labor, the greater the consequences for newborns. In the present study, it was observed that the longer the dystocia, the greater the implications for the placenta and, potentially, for the neonates. Therefore, early obstetric intervention is essential to safeguard maternal health and to maximize neonatal survival.

One potential limitation of this study is that the owners reported the length of dystocia during the anamnesis, and this information may not be entirely accurate. Another possible limitation is the number of cases in some groups. The lack of independence among multiple placentas from the same individual could be another limitation. In addition, there are residual confounding factors related to breed, litter size, the mother’s health status, and anesthetic management. Another limitation was the lack of blood biochemistry data, which prevented the evaluation of systemic maternal or fetal alterations associated with prolonged dystocia. However, it is important to note that all the bitches survived after the surgery and no perioperative deaths were recorded. The principal limitation in canine neonatal studies is the difficulty in obtaining samples before, during, and after birth. Despite these limitations, the results presented support our hypothesis, demonstrating that prolonged hypoxia increases the degree of placental injury with potential consequences for neonatal survival and postnatal development. In addition, our findings provide new insights into the pathological consequences in the placentas from dystocia, which could be helpful in future studies on growth, metabolism, and behavior in newborns from prolonged deliveries.

## 5. Conclusions

The infiltration of polymorphonuclear cells and macroscopic congestion, observed to a severe degree in prolonged dystocia in this study, suggest that the duration of dystocia is associated with the presence and severity of placental injuries, consistent with a prolonged hypoxia model. The results of this work indicate the need for early obstetric intervention in all cases of dystocia to minimize fetal hypoxia and improve neonatal outcomes. The placenta may serve as a biomarker of neonatal compromise; the presence and severity of lesions may indicate a higher risk of reduced survival and require closer monitoring during the perinatal period. Moreover, these findings may be of comparative interest to other species. Further prospective studies with larger sample sizes are needed to confirm these associations and clarify their clinical implications.

## Figures and Tables

**Table 1:** Characteristics of bitches included in this study, categorized by dystocia duration groups.

^1^ Group	^2^ Breeds (n)	^3^ Number of Bitches (n)	^4^ Age (Years, Mean ± SD)	^5^ BCS (1–5)	^6^ Primiparous n (%)
A	Poodle (2)	3	3.33 ± 0.58	3–4	3 (100%)
	Golden (1)				
B	French Bulldog (2)	4	2.62 ± 1.60	3–4	3 (75%)
	Chihuahua (1)				
	Dachshund (1)				
C	Mixed-breed (4)	7	7.21 ± 2.00	2–4	4 (57%)
	Poodle (2)				
	Pinscher (1)				
D	Mixed-breed (1)	4	2.62 ± 1.60	3–4	3 (75%)
	Pitbull (1)				
	French Bulldog (2)				

^1^ Group: Based on dystocia duration (A, up to 6 h; B, 6–11.9 h; C, 12–24 h; D, more than 24 h); ^2^ breeds (n): breed included in each group with the corresponding number of animals; ^3^ number of bitches (n): total number of bitches in each group; ^4^ age: expressed as mean ± SD (standard desvio) for each group; ^5^ BCS: body condition score on scale of 1 to 5; ^6^ primiparous n (%): number and percentage of primiparous bitches in each group.

**Table 2:** Logistic regression models assessing the effect of dystocia duration (in hours), as a quantitative predictor, on the risk of having placental lesions in cases of canine dystocia (n: 47 placentas from 18 bitches).

^1^ Effect	^2^ Lesion (n)	^3^ OR	^4^ 95% CI	*p*
		^5^ Risk of macroscopic congestion		
Dystocia duration	No (17)	Ref.		
(increment of 1 h over the mean)	Yes (30)	1.08	0.94–1.24	0.26
		^6^ Risk of macroscopic necrosis		
Dystocia duration	No (27)	Ref.		
(increment of 1 h over the mean)	Yes (20)	1.11	0.98–1.26	**0.09**
		^7^ Risk of microscopic congestion		
Dystocia duration	No (5)	Ref.		
(increment of 1 h over the mean)	Yes (42)	1.01	0.87–1.16	0.90
		^8^ Risk of microscopic necrosis		
Dystocia duration	No (17)	Ref.		
(increment of 1 h over the mean)	Yes (30)	0.99	0.90–1.09	0.90
		^9^ Risk of infiltration		
Dystocia duration	No (17)	Ref.		
(increment of 1 h over the mean)	Yes (30)	1.09	0.97–1.25	0.14
		^10^ Risk of mineralization		
Dystocia duration	No (41)	Ref.		
(increment of 1 h over the mean)	Yes (6)	1.10	0.99–1.23	**0.06**

^1^ Effect: quantitative effect of dystocia duration (hours). ^2^ Lesion (n): number of placentas with presence of lesions (no vs. yes). ^3^ OR: odds ratio. The odds were expressed per unit (h) increment above the mean dystocia duration (mean: 12 h). ^4^ 95%CI: confidence interval of the odds ratio. ^5^ Risk of macroscopic congestion: presence of violet-colored areas visible in the placental girdle. ^6^ Risk of macroscopic necrosis: presence of yellow ochre-colored areas visible in the placental girdle. ^7^ Risk of microscopic congestion: presence of excessive accumulation of blood within placental blood vessels. ^8^ Risk of microscopic necrosis: loss of structural detail in placental areas, accompanied by eosinophilic cytoplasm and nuclear changes, including pyknosis, karyorrhexis, and karyolysis, or by the absence of nuclei. ^9^ Risk of inflammatory infiltrate: presence of polymorphonuclear leukocyte cells. ^10^ Risk of mineralization: presence of basophilic stippling in necrotic cells, indicative of calcium salt deposition. *p* < 0.05 indicates significant differences, and *p*-values between 0.05 and 0.10 indicate a statistical trend and are shown in bold.

**Table 3:** Multinomial regression models assessing the effect of dystocia duration (in hours), as a quantitative predictor, on the severity of placental lesions in cases of canine dystocia (n: 47 placentas from 18 bitches).

^1^ Effect	^2^ Grades (n)	^3^ OR	^4^ 95% CI	*p*
Dystocia duration		^5^ Risk of macroscopic congestion		
(increment of 1 h over the mean)	0 (17)	Ref.		**0.01**
	1 (6)	0.62	0.39–0.99	
	2 (14)	1.08	0.84–1.38	
	3 (10)	1.44	1.05–1.97	
Dystocia duration		^6^ Risk of macroscopic necrosis		
(increment of 1 h over the mean)	0 (27)	Ref.		0.16
	1 (7)	1.13	0.98–1.30	
	2 (6)	1.04	0.87–1.24	
	3 (7)	1.16	1.01–1.34	
Dystocia duration		^7^ Risk of microscopic congestion		
(increment of 1 h over the mean)	0 (5)	Ref.		0.48
	1 (23)	0.97	0.83–1.14	
	2 (19)	1.04	0.89–1.20	
Dystocia duration		^8^ Risk of microscopic necrosis		
(increment of 1 h over the mean)	0 (17)	Ref.		0.83
	1 (24)	0.98	0.88–1.09	
	2 (6)	1.01	0.89–1.15	
Dystocia duration		^9^ Risk of infiltration		
(increment of 1 h over the mean)	0 (17)	Ref.		**0.06**
	1 (20)	1.05	0.90–1.21	
	2 (10)	1.18	1.01–1.39	
Dystocia duration		^10^ Risk of mineralization		
(increment of 1 h over the mean)	0 (41)	Ref.		0.15
	1 (1)	1.16	0.95–1.41	
	2 (5)	1.09	0.97–1.22	

^1^ Effect: quantitative effect of dystocia duration (hours). ^2^ Grades (n): number of placentas having different grades of lesions. Macroscopic congestion and necrosis were evaluated using a 4-point scale, where 0 = no alteration, 1 = mild alteration, 2 = moderate alteration, and 3 = severe alteration [2,11]. Microscopic necrosis, congestion, mineralization, and infiltration of polymorphonuclear cells were evaluated using a 3-point scale, where 0: no lesion, 1: up to 40% of the evaluated field affected, 1–3 lesions; and 2: more than 40% of the field affected, >3 lesions [2,11]. ^3^ OR: odds ratio. The odds were expressed per unit (h) increment above the mean dystocia duration (mean: 12 h). ^4^ 95%CI: confidence interval of the odds ratio. ^5^ Macroscopic congestion: presence of violet-colored areas visible in the placental girdle. ^6^ Macroscopic necrosis: presence of yellow ochre-colored areas visible in the placental girdle. ^7^ Microscopic congestion: presence of excessive accumulation of blood within placental blood vessels. ^8^ Microscopic necrosis: loss of structural detail in placental areas, accompanied by eosinophilic cytoplasm and nuclear changes, including pyknosis, karyorrhexis, and karyolysis, or by the absence of nuclei. ^9^ Inflammatory infiltrate: presence of polymorphonuclear leukocyte cells. ^10^ Mineralization: presence of basophilic stippling in necrotic cells, indicative of calcium salt deposition. *p* < 0.05 indicates significant differences, and *p*-values between 0.05 and 0.10 indicate a statistical trend and are shown in bold.

## Data Availability

The original contributions presented in this study are included in the article. Further inquiries can be directed at the corresponding author.

## Supplementary Materials

The following supporting information can be downloaded at: https://www.mdpi.com/article/10.3390/life16020349/s1, Table S1. Complete information per-placenta raw variables (group, breed, parity, lesion grades, and puppy outcome).
