# Supplementary material for: Effect of Dystocia Duration on the Placental Health in Canines
Source: Life (Basel). 2026 Feb 18;16(2):349. doi: 10.3390/life16020349 (PMC12941726; doi:10.3390/life16020349)
Supplement: Supplementary file 1 [file life-16-00349-s001.zip › life-4041484-supplementary.pdf]

Table S1. Complete information per-placenta raw variables (group, breed, parity, lesion grades, and puppy outcome).

[1]Pla ID	[2]M ID	[3]Group	[4]Breed	[5]Parity	[6]Puppy outcome	[7]CMa	[8]NMa	[9]CMi	[10]NMi	[11]Min	[12]PMN
P1	M1	A	Poodle	P	Stillborn	0	0	1	1	0	1
P2	M1	A	Poodle	P	Stillborn	1	0	1	1	0	0
P3	M1	A	Poodle	P	Stillborn	1	0	1	1	0	0
P4	M1	A	Poodle	P	Stillborn	1	0	1	0	0	0
P5	M2	A	Poodle	P	Stillborn	0	0	1	0	0	1
P6	M2	A	Poodle	P	Alive	1	0	1	0	0	0
P7	M3	A	Golden	P	Stillborn	2	0	2	2	0	2
P8	M4	B	French Bulldog	M	Alive	0	0	1	0	0	0
P9	M4	B	French Bulldog	M	Alive	0	0	1	0	0	1
P10	M4	B	French Bulldog	M	Alive	0	0	1	0	0	0
P11	M4	B	French Bulldog	M	Alive	0	0	1	0	0	0
P12	M4	B	French Bulldog	M	Alive	0	0	1	0	0	1
P13	M5	B	Chihuahua	P	Stillborn	0	0	2	1	0	2
P14	M6	B	Dachshund	P	Stillborn	0	0	2	1	0	1
P15	M6	B	Dachshund	P	Alive	0	0	1	1	0	1
P16	M6	B	Dachshund	P	Alive	0	0	2	1	0	1
P17	M7	B	French Bulldog	P	Stillborn	2	2	1	1	0	1
P18	M7	B	French Bulldog	P	Stillborn	2	1	1	2	0	1
P19	M7	B	French Bulldog	P	Stillborn	2	0	2	2	0	2
P20	M7	B	French Bulldog	P	Stillborn	2	0	1	1	0	1
P21	M7	B	French Bulldog	P	Alive	2	0	2	2	0	2
P22	M8	C	Mixed	P	Stillborn	3	3	0	1	2	0
P23	M8	C	Mixed	P	Stillborn	2	2	2	1	2	1
P24	M8	C	Mixed	P	Stillborn	2	2	0	1	2	1
P25	M8	C	Mixed	P	Stillborn	2	2	2	1	0	1
P26	M9	C	Mixed	P	Alive	0	0	1	1	0	1
P27	M9	C	Mixed	P	Alive	0	0	2	1	0	1
P28	M9	C	Mixed	P	Alive	0	0	2	1	0	1
P29	M9	C	Mixed	P	Stillborn	0	0	2	1	0	1
P30	M10	C	Poodle	M	Stillborn	0	3	1	1	0	0
P31	M10	C	Poodle	M	Stillborn	0	3	1	1	0	1
P32	M11	C	Mixed	P	Stillborn	2	2	2	0	0	0
P33	M11	C	Mixed	P	Stillborn	2	3	2	0	0	0
P34	M11	C	Mixed	P	Stillborn	2	2	2	0	0	0
P35	M12	C	Mixed	M	Alive	1	0	0	0	0	0
P36	M12	C	Mixed	M	Alive	1	1	0	0	0	0
P37	M12	C	Mixed	M	Alive	2	1	0	0	0	0
P38	M13	C	Pinscher	M	Stillborn	3	1	1	1	0	2
P39	M14	C	Poodle	P	Stillborn	3	1	1	1	0	1
P40	M14	C	Poodle	P	Alive	3	0	2	1	0	1
P41	M14	C	Poodle	P	Alive	3	1	1	0	0	0
P42	M15	D	Mixed	P	Stillborn	2	0	2	0	0	2
P43	M16	D	French Bulldog	M	Stillborn	3	3	1	1	1	1
P44	M16	D	French Bulldog	M	Stillborn	3	3	2	0	2	2
P45	M17	D	Pitbull	P	Stillborn	3	3	2	2	2	2

P46	M18	D	French Bulldog	P	Stillborn	3	1	1	2	0	2
P47	M18	D	French Bulldog	P	Stillborn	3	0	2	1	0	2

[1]Pla ID: Placental Identification; [2]M ID: Maternal identification; [3]Group: Group based on duration; [4]Breed: Breed of each animal; [5]Parity: primiparous (P) vs multiparous (M); [6]Puppy outcome: Stillborn or alive; [7]CMa: macroscopic congestion; [8]NMa: macroscopic necrosis; [9]CMi: microscopic congestion; [10]NMi: microscopic necrosis; [11]Min: mineralization; [12]PMN: inflammatory infiltrate.
